# Editorial Note

**Published:** 1892-03

**Authors:** R. Shingleton Smith


### Editorial Note.

The readers of the *Journal* cannot fail to observe on the cover of this number an important change, which needs a few words of comment.

When the first number was issued in 1883, it had yet to be proved whether the demand for such a *Journal* would be equal to the expenditure which would necessarily be entailed: the Bristol Medico-Chirurgical Society made itself responsible for any possible deficiency, and has year by year been rewarded by a handsome surplus, which is now being utilised in the establishment of a medical library for Bristol and the West.

This result is doubtless due to the literary qualities of the *Journal* itself in conjunction with the able management it has received from the editorial and publishing staff.

The retiring Editor may be congratulated on the position which his *Journal* has obtained in the tenth year of its life: it has passed through the perils of the infantile period, and is now thriving on an established reputation. At this stage he leaves it; he has given to it nearly a decade of active supervision and original genius, and has sown the seeds which should develop into even greater products in the future. The members of the Bristol Medico-Chirurgical Society and the subscribers to the *Journal* will doubtless join in the resolution which has been adopted by the Committee of the Society expressing their regret that Mr. J. Greig Smith finds it necessary to resign his office of Editor, and their cordial thanks to him for all that he has done towards the encouragement and development of literary talent in this neighbourhood. They will wish him a continuance of that success which he has already achieved in other literary fields, and will hope to still have his assistance in the maintenance of his own offspring.

As one of the original founders of the Society under whose auspices the *Journal* is published, and its Secretary for the first fourteen years, I have naturally taken a keen interest in the welfare of all its branches, and I have been gratified at the steady expansion of its scope far beyond the limits at first contemplated. I have not been hitherto officially connected with the *Journal* staff, and therefore have no necessity on the ground of modesty to withhold commendation where it is due; I may therefore freely state my conviction that few societies have better occasion to take a pride in the position which they hold and the advantages which they give. These are to a great extent due to the *Journal* itself, which directly or indirectly has made the establishment of a really good and cosmopolitan medical library a possibility, and for which a home has been found in a Club replete with every comfort.

I hope to carry on the work on the lines on which the *Journal* is now working so satisfactorily. No radical reforms are contemplated. With the assistance of the same Editorial Committee, and of the same indefatigable Assistant-Editor, I believe that the literary repute obtained by the personal and powerful influence of my predecessor can be maintained, and no effort on my part will be wanting to render the *Journal* even more worthy of support in the future than it has been in the past.

R. SHINGLETON SMITH.

